# Impact of Harvest Conditions and Host Tree Species on Chemical Composition and Antioxidant Activity of Extracts from *Viscum album* L.

**DOI:** 10.3390/molecules26123741

**Published:** 2021-06-19

**Authors:** Wioleta Pietrzak, Renata Nowak

**Affiliations:** Department of Pharmaceutical Botany, Medical University of Lublin, Chodźki 1 Str., 20-093 Lublin, Poland; renata.nowak@umlub.pl

**Keywords:** *Viscum album*, LC-MS analysis, polyphenols, antioxidant activity

## Abstract

The content of plant secondary metabolites is not stable, and factors such as the region/location effect and seasonal variations have an impact on their chemical composition, especially in parasitic plants. Research in this area is an important step in the development of quality parameter standards of medicinal plants and their finished products. The effects of the time and place of harvest and the host tree species on the chemical composition and antioxidant activity of mistletoe extracts were investigated. Statistical tools were used to evaluate the results of the spectrophotometric and LC-ESI-MS/MS studies of the phenolic composition and antioxidant activity. The investigations indicate that the qualitative and quantitative composition, influencing the biological activity of mistletoe extracts, largely depends on the origin of the plant. The mistletoe extracts exhibited a rich phenol profile and high antioxidant activity. The chemometric analysis indicated that mistletoe collected from conifers (*Viscum abietis* and *Viscum austriacum*) had the most advantageous chemical composition and antioxidant activity. Moreover, the chemical profile and biological activity of the plant material were closely related to the climatic conditions and location of the harvested plant. Higher levels of phenolic compounds and high antioxidant activity were found in extracts obtained from plant material collected in cold weather with the presence of snow and less sunshine (autumn–winter period).

## 1. Introduction

*Viscum album* L. (Loranthaceae), commonly known as mistletoe, is an evergreen hemi-parasitic plant widely distributed throughout the globe. This amazing plant lives at the expense of its hosts, i.e., various tree species. Chemical and pharmacological studies of mistletoe have identified different kinds of constituents such as viscotoxins, phenylpropanes, lignans, flavonoids, amines, polysaccharides, and lectins (ML1, ML2, ML3, and ML4) [1]. Some of them, especially flavonoids and phenolic acids, are natural antioxidants involved in the biological activity of the plant. The biological activity of *Viscum*, such as antioxidant, anti-inflammatory, and antibacterial properties as well as antiepileptic, immunostimulatory, and antiviral activity, have been reported [2,3,4,5,6,7]. One of the most important uses of mistletoe extracts is cancer therapy, including breast cancer [3,8], pancreatic cancer [9], bladder cancer [10], laryngeal cancer [11], lymphoblastic leukemia [12], and on medulloblastoma cells [13]. Water extracts from fresh mistletoe exert their anticancer effects by multifarious ways—immune augmentation, tumor prevention, malignant tissue inhibition, the moderation of chemotherapeutics side effects and DNA protection. The most active ingredients of these extracts are lectins and viscotoxins, whose content are not stable. Research in this area indicates species variability and seasonal fluctuations of the composition of mistletoe. The mistletoe-based formulations such as Iscador, Isorel, Iscucin, Lektinol, Eurixor, Helixor, Abnoba-viscum and recombinant lectin ML-1 have been approved for commercial use [14,15].

The recent literature also provides reports on the hypoglycemic effect of mistletoe preparations [6,16,17,18]. However, due to the diversity of products from mistletoe, which is a parasitic plant, and the proportions of pharmacologically relevant constituents, the interpretation of clinical research is difficult.

The results of many studies on the content of phenolic metabolites in medicinal plants have confirmed the hypothesis that these compounds significantly contribute to the antioxidant activity. The literature reports beneficial effects of antioxidants in the chemoprevention of various diseases caused by/or related to oxidative stress induced by excess free radicals in the human body. These diseases include cancer, hypertension, heart disease, and diabetes [19]. In particular, the use of antioxidants in the treatment of diabetes is extremely important. Oxidative stress contributes to the loss of β-cell function, the aggravation of insulin resistance, and the development of vascular complications. In patients with diabetes, the level of oxidative damage increases with the severity of the disease [20].

The content of secondary plant metabolites is not stable. Studies have shown that the content of phenolic compounds may vary depending on biotic and abiotic factors, the growth stage, the part of the plant, and the characteristics of the environment [21,22,23]. Most herbal medicines produced currently in a majority of developing countries lack proper quality specification and standards. The quality of the finished product is based on the quality of raw materials, which also depends on various genetic, ontogenic, morphogenetic, and environmental factors. These factors influence the biosynthesis of secondary plant metabolites and fluctuation in their contents. These facts should be taken into account while developing quality parameter standards of medicinal plants and their finished products [24,25]. Especially in the case of parasitic plants, factors such as the region/location effect and seasonal variation have an impact on the chemical composition of the plant.

Mistletoes are leafless flowering parasitic plants that kill by slowly robbing the tree of food and water. Their infections can retard growth and reduce seed production and wood quality. Heavy, long-term infections can kill trees. Although mistletoe infections are a common problem in many countries, the plant has a rich chemical composition. Mistletoe can biosynthesize its own compounds or can take some nutrients from the host trees. It has been suggested that the phytochemical profile of *Viscum* depends on the host of this parasitic plant [26,27,28,29,30]. The chemical composition and biological activity of mistletoe is not stable and may be associated with environmental conditions, such as temperature, carbon dioxide concentration, pollution, soil fertility, and season of the year [31].

The aim of the present study was to investigate the effect of the time and place of harvest and the host tree species on the chemical composition and antioxidant activity of mistletoe extracts. Moreover, in the current work, chemometric analysis of the results of the spectrophotometric and LC-ESI-MS/MS studies and climatic conditions was performed. It should be emphasized that this study is the first of such a comprehensive analysis of the impact of climatic factors, place of harvest, and host tree on the quality of harvested *Viscum* herb.

## 2. Results and Discussion

Mistletoe is a chemical-rich plant. Studies carried out so far have shown the presence of a wide variety of compounds responsible for the properties and biological activity of the genus *Viscum* L. Phenolics and flavonoids present in mistletoe are largely responsible for its biological effects, including anti-radical activity. Therefore, it is important to develop a method for the acquisition of extracts with a high content of biologically active compounds [7,32,33]. For a long time, scientists have been interested in the issue of the impact of the host on the chemical composition in mistletoe. Previous studies in this area are often contradictory and ambiguous in this regard, suggesting that the species of tree parasitized by mistletoe may partially determine the specific synthesis of some secondary metabolites [26,27,28,29,30]. The variability of the chemical composition and biological activity of mistletoe can also be associated with environmental conditions, i.e., ambient temperature, carbon dioxide concentration, pollution, soil fertility, and season [31].

### 2.1. Effect of the Harvest Season on the Chemical Composition and Antioxidant Activity of Mistletoe Extracts (Viscum album subsp. album)

In this part of the study, changes in the composition of polyphenols and flavonoids and antioxidant activity depending on the time of mistletoe harvest were examined.

The impact of the mistletoe collection period on the chemical composition and biological activity of extracts was studied previously by Vicas et al. (2011) and Önay-Uçar et al. (2006). The authors studied the TPC and antioxidant activity of aqueous and methanolic extracts from leaves and stems of mistletoe in three periods (May, July, and December) [33] and the antioxidant activity of methanolic extracts from *Viscum album* leaves harvested in February and July [7]. They found that the differences in the antioxidant activity of mistletoe harvested from different trees and in different seasons can be attributed to factors such as climate and temperature, which can significantly affect the chemical composition and antioxidant activity of plants [7,33]. However, the authors did not refer their results to climatic conditions (e.g., temperature) and places of harvesting the plant material for testing; hence, the data in this area are insufficient.

Therefore, preliminary screening research was performed to determine the effect of climatic factors and raw material harvest time on the chemical composition and antioxidant activity of mistletoe extracts. To the best of our knowledge, no such thorough research in this topic has been conducted.

In this study, plant material was collected from the same host tree species (*Populus nigra* L.) and from the same harvesting place (Olszowiec), i.e., factors that could affect the results were eliminated.

**Figure 1:**
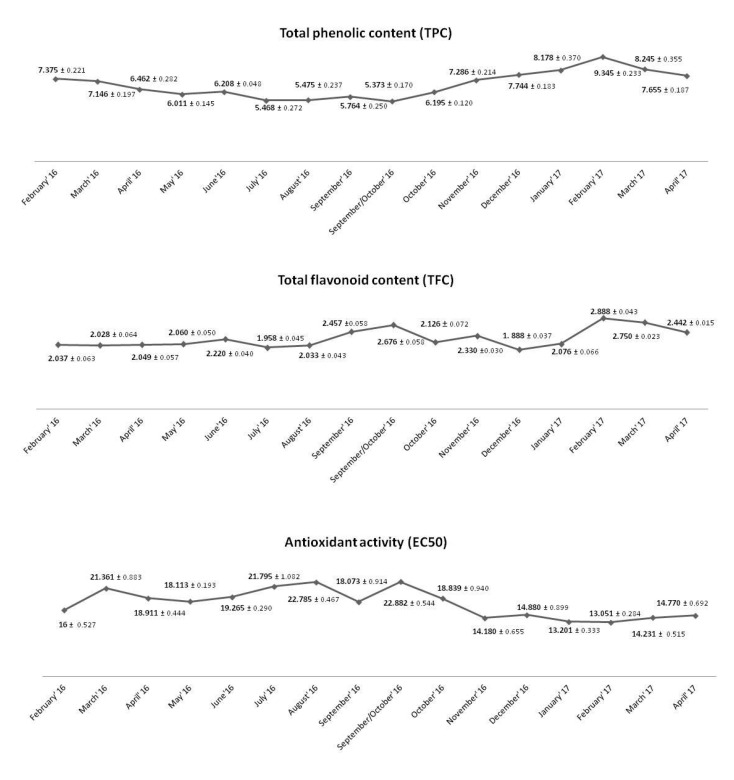
Total phenolic content (TPC; mg GA/g of dry extract), flavonoid content (TFC; mg Q/g of dry extract) and antioxidant activity (EC_50_; mg dry extract/mg of DPPH) of methanol extracts from mistletoe (from *Populus nigra* L.) harvested at various time in Olszowiec region.

The results of total phenolic content (TPC), flavonoid content (TFC), and EC_50_ values of extracts from mistletoe harvested at different times from *Populus nigra* L. are shown in Figure 1.

The TPC value varied between 7.146 and 9.345 mg GA g^−1^ of dry extract, TFC—from 1.888 to 2.888 mg Q g^−1^ of dry extract, and EC_50_ values from 13.051 to 21.361 mg dry extract mg^−1^ of DPPH^•^. The results obtained in the present study showed differences between the harvest seasons, with the highest amount of polyphenols and flavonoids and high antioxidant activity in extracts from mistletoe collected in the autumn–winter period (November–March).

The study included data on the meteorological conditions prevailing during the harvest time in the Olszowiec region, which are shown in Table 1. Moreover, statistical analysis of the results was performed and Spearman correlation coefficients were calculated (Table 2).

Based on the results of spectrophotometric analysis, it can be seen that the extracts from the plant material collected in February, March, and April 2017 contained more polyphenols and flavonoids and had higher antiradical activity than the samples obtained from mistletoe harvested in February, March, and April in 2016. As regards the meteorological conditions recorded during the harvest time in the Olszowiec region, it can be concluded that the improved properties of the extracts from 2017, compared to those from 2016, are related to:

- the lower average air temperature in February–April 2017;
- the presence, thickness, and deposition time of snow cover in February–April 2017;
- lower sunshine duration in February–April 2017.

Moreover, based on the results of the statistical analysis of the total phenolic and flavonoid content, EC_50_ values, and climatic conditions, it can be concluded that:

- there was a strong relationship between TPC, EC_50_, and the average air temperature. The correlation coefficients were 0.8355 and 0.7466, respectively;
- the total phenolic content and antioxidant activity largely depended on the maximum snow depth, maximum persistence of snow cover, and the number of days with snow. The correlation coefficients varied between 0.7178 and 0.8341;
- there was a low correlation between the values of TFC and climatic conditions prevailing during the harvest of the plant material.

### 2.2. Analysis of the Phenolic Profile of Mistletoe Extracts Depending on the Place of Harvesting the Plant Raw Material

In this part of the study, the effect of the harvesting place on the similarity of the phenolic profile of mistletoe extracts harvested from apple trees (*Malus domestica* Borkh.) was examined. For this purpose, spectrophotometric screening tests (total phenolic content, total flavonoid content, and antioxidant activity measured with the DPPH^•^ method) and LC-MS identification of phenolic acids (LC-ESI-MS/MS) in the mistletoe extracts were carried out (Table 3 and Table 4).

The spectrophotometric studies showed slight differences in the total content of phenolics (from 33.789 mg GA per g of dry extract for sample T5 to 48.628 mg GA g^−1^ of dry extract for sample T2), the content of flavonoids (from 11.722 mg Q g^−1^ of dry extract for sample T3 to 13.786 mg Q g^−1^ of dry extract for sample T1), and antioxidant activity (from 3.753 mg dry extract per mg of DPPH^•^ for sample T3 to 4.608 mg dry extract per mg of DPPH^•^ for sample T1).

However, more diverse results were observed in the spectrometric studies of phenolic acids. The higher differences in the content of acids in extracts from the mistletoe harvested from apple trees were observed in the case of protocatechuic (from 64.60 µg per g of dry extract for sample T3 to 233.44 µg per g of dry extract for sample T5), syringic (from 21.40 µg per g of dry extract for sample T2 to 57.91 µg per g of dry extract for sample T4), caffeic (from 46.06 µg per g of dry extract for sample T1 to 72.95 µg per g of dry extract for sample T4), and synapinic (from 15.05 µg per g of dry extract for sample T5 to 68,87 µg per g of dry extract for sample T4) acids.

The quantitative results of phenolic acids in mistletoe extracts collected from five different apple trees (from five different areas of the Lublin region—Supplementary Table S1) and the spectrophotometric results were subjected to chemometric analysis. The calculations were performed in Statistica version 10.0.

Compliance with the normal distribution of individual parameters was determined using the Shapiro–Wilk test. The data were subsequently standardized and cluster analysis was performed for the data. For this purpose, one method of cluster analysis was applied: k-means clustering. In general, a k-means algorithm groups methods in clusters of the greatest possible distinction. The results of the statistical analysis are shown in Supplementary Figure S1.

In this part of the research, the host tree species (*Malus domestica* Borkh.) and the mistletoe harvesting time (November 2017) were the same for each of the tested T1–T5 extracts (each extract was an average sample of 10 apple trees from one orchard); therefore, this study eliminated the impact of these factors on the chemical composition of mistletoe. It can be assumed that the qualitative and quantitative chemical composition of extracts is affected by the place of harvesting of the plant raw material, which is confirmed by previous theoretical literature reports. To the best of our knowledge, there is no experimental research on the impact of the raw material harvesting site on the chemical composition in mistletoe. Our tests are preliminary screening experimental research in this field, and for further and deeper analysis is necessary.

In summary, based on the spectrophotometric screening tests, it can be seen that the extracts obtained from mistletoe parasitizing *Malus* Mill. (T1–T5) did not show significant differences in the TPC, TFC, and EC_50_ values, as they originated from the same host species—*Malus domestica* Borkh.

In addition, the statistical analysis shows that the place of harvesting of plant material is an additional factor that can affect the diversity of the chemical composition of extracts determined with the LC-MS method.

### 2.3. Analysis of the Phenolic Profile of Mistletoe Extracts Depending on the Host Tree Species

As mentioned previously, the chemical composition and antioxidant activity of mistletoe extracts are not stable and depend on various factors. In the case of hemi-parasitic plants, this problem should be considered in the context of their habitats, i.e., the influence of the host trees on the chemical composition of the plant.

Therefore, after assessing the impact of climatic conditions and the time and place of harvesting of the plant material, we decided to determine the possible relationships between the chemical composition and antioxidant activity of mistletoe and the host tree species from which the plant was harvested.

Comparative spectrophotometric studies of methanolic extracts from mistletoe were performed to determine the content of phenolic compounds (TPC, TFC) and antioxidant activity. The results are presented in Table 5.

The results show that the total phenolic content ranged from 30.56 ± 1.76 mg GA g^−1^ of dry extract for mistletoe extract from *Fraxinus exscelsior* L. (*Viscum album* subsp. *album*) to 56.75 ± 1.31 mg GA per g of dry extract for mistletoe extract from *Abies alba* Mill. (*Viscum album* subsp. *abietis*). TFC varied from 10.11 ± 0.42 mg Q g^−1^ of dry extract (for mistletoe extract from *Abies alba* Mill.) to 16.90 ± 0.32 mg Q g^−1^ of dry extract (for mistletoe extract from *Pinus sylvestris* L.—*V. austriacum*).

The antioxidant activity was measured using the DPPH^•^ scavenging assay, antiradical capacity determination with ABTS^+•^, and the Oxygen Radical Absorbance Capacity (ORAC) assay.

The lowest EC_50_ value, i.e., the highest antioxidant activity determined with the DPPH^•^ method, was observed in the mistletoe extract obtained from *Acer saccharinum* L. (2.14 ± 0.09 mg dry extract mg^−1^ of DPPH^•^). The mistletoe extracts from *Malus domestica* Borkh., *Fraxinus exscelsior* L., and *Sorbus aucuparia* L. had the lowest antioxidant activity (EC_50_ of 4.05 ± 0.19, 4.21 ± 0.11, and 4.35 ± 0.08 mg dry extract per mg of DPPH^•^, respectively).

In the ABTS^+●^ method, the highest antioxidant activity was exhibited by the extract from mistletoe harvested from *Abies alba* Mill. (0.21 ± 0.08 mM Trolox per g dry extract).

In the ORAC method, the extracts from mistletoe collected from conifers (*Viscum album* subsp. *abietis* and *Viscum album* subsp. *austriacum*) were the most active samples (1.95 ± 0.10 and 2.01 ± 0.10 mM Trolox per g dry extract, respectively).

In order to show differences and similarities in the qualitative and quantitative chemical composition of mistletoe extracts from various host trees, the simple, rapid, reliable, and effective LC-ESI-MS/MS method was used. As shown by the available literature data, the quantitative and qualitative determinations of 14 phenolic acids and 16 flavonoid aglycones in three mistletoe subspecies collected from 12 host trees were performed for the first time (Table 6 and Table 7). The chromatograms of phenolic acids and flavonoid aglycones for exemplary samples are shown in the Supplementary Materials in Figures S2 and S3.

As indicated by the results of the quantitative and qualitative studies of free phenolic acids in the methanolic extracts from *Viscum album* L., no veratric acid and 3-OH-cinnamic acid were found in the analyzed extracts. Rosmarinic and gentisic acids were detected only in trace amounts, depending on the sample tested. Gallic acid was present in small amounts in the tested samples from the raw material collected from *Fraxinus exscelsior* L. (46.62 μg/g dry extract) and *Tilia cordata* Mill. (33.71 μg/g extract).

Protocatechuic acid was mainly present in the mistletoe extract collected from *Malus domestica* Borkh. (118.03 ± 0.86 μg/g extract), *Fraxinus pensylvanica* Marsh. (133.27 ± 1.20 μg/g extract), *Fraxinus exscelsior* L. (203.48 ± 9.16 μg/g of extract), *Tilia cordata* Mill. (138.17 ± 1.17 μg/g extract), and *Abies alba* Mill (114.29 ± 1.05 μg/g extract).

Vanillic acid was detected in all tested extracts in various amounts: from trace to large amounts for the mistletoe extracts from *Populus nigra* L. and *Populus nigra* L. ‘*Italica*’ and conifer mistletoe (*V. album* subsp. *abietis* and *V. album* subsp. a*ustriacum*).

4-hydroxybenzoic acid, which was most abundant in *V. album* subsp. *abietis* (164.91 ± 4.95 μg/g of extract), was a characteristic phenolic acid as well.

In the case of *p*-coumaric and syringic acids, the highest content was found in *Populus nigra* L. ‘*Italica*’ and conifer mistletoe (*V. album* subsp. *abietis* and *V. album* subsp. a*ustriacum*). In turn, salicylic acid was a characteristic compound for *V. album* subsp. *austriacum* and mistletoe extracts from *Populus nigra* L. and *Populus nigra* L. ‘*Italica*’.

The analysis of the content of aglycones in the tested samples revealed the absence of compounds such as morin, chrysin, and prunetine, while taxifolin was present only in some samples in trace amounts.

Myricetin was found in the highest amounts in the mistletoe extract from *Malus domestica* Borkh. (1.91 ± 0.006 μg/g of extract). Eriodictiol significantly predominated in *V. album* subsp. *album* from *Tilia cordata* Mill., *Fraxinus pensylvanica* Marsh., and *Fraxinus exscelsior* L. Quercetin was determined only in *V. album* subsp. a*ustriacum* and *V. album* subsp. *album* from *Malus domestica* Borkh., whereas kaempferol was detected in *V. austriacum* and *V. album* from *Acer platanoides* L.

Extracts from *V. austriacum* also contained large amounts of sakuranetin, i.e., 14.44 ± 0.14 μg/g of extract, and extracts from *V. album* harvested from *Acer platanoides* L. had large amounts of apigenin (57.32 ± 0.15 μg/g of extract).

Isoramnetin, rhamnetin, and rhamnazine were the most characteristic compounds in the mistletoe from *Viscum austriacum* and *Viscum album* from *Malus domestica* Borkh.

The highest amount of phenolic acids was determined with the LC-MS method in *Viscum album* subsp. *abietis* (601.45 μg/g of extract), and the highest content of flavonoid aglycones were detected in *Viscum album* subsp. *austriacum* (56.08 μg/g of extract) and *Viscum album* from *Malus domestica* Borkh. (57.14 μg/g of extract).

**Figure 2:**
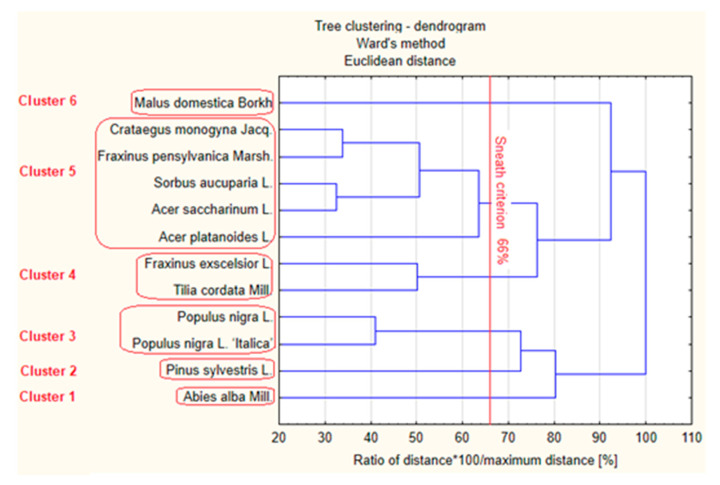
Dendrogram showing similarity of mistletoe extracts obtained from different host trees with a calibrated axis of the binding distance and with the 66% criterion of Sneath.

The results of the spectrophotometric assays and LC-MS analysis of phenolic compounds in the methanolic extracts from *Viscum* collected from various host tree species were subjected to chemometric analysis. The calculations were performed in Statistica version 10.0. Chemometrics facilitates understanding of chemical information obtained and correlates quality parameters or biological properties with analytical data. In this part of the study, the joining (tree-clustering) method of cluster analysis was applied. In the tree clustering, the Ward’s method was used for amalgamation, and the Euclidean distance was adopted as a measure of distance between the clusters. The results of the statistical analysis, i.e., the hierarchical tree dendrogram, are shown in Figure 2. The tree dendrogram shows clusters of objects according to the strength of their correlation. This correlation can be seen in the expression profiles of objects from the same cluster.

Based on the dendrogram, six groups showing relative similarity among the chemical composition in *Viscum album* L. can be identified according to the less restrictive Sneath’s criterion (66%). Figure 2 shows two separate clusters (cluster 1 and 2) with extracts of mistletoe harvested from conifers (*Viscum abietis* from *Abies alba* Mill. and *Viscum austriacum* from *Pinus sylvestris* L). A separate cluster 3 is also formed by extracts of mistletoe from poplar (*V. album* from *Populus nigra* L. and *Populus nigra* L. ‘*Italica*’). Cluster 4 contains extracts of mistletoe from *Fraxinus exscelsior* L. and *Tilia cordata* Mill., while cluster 5 includes mistletoe extracts from *Crataegus monogyna* Jacq., *Fraxinus pensylvanica* Marsh., *Sorbus aucuparia* L. and two subspecies of maple (*Acer saccharinum* L. and *Acer platanoides* L.). Mistletoe extracts from apple trees (*Malus domestica* Borkh.) constitute a separate cluster 6. The extracts within a given cluster show similarities in the polyphenol profile and antioxidant activity.

The chemometric analysis shows that mistletoe derived from conifers (*Viscum album* subsp. *abietis* and *Viscum album* subsp. *austriacum*) is the most advantageous material in terms of the chemical composition and antioxidant activity.

As shown by the available literature data, the analysis of phenolic acids in mistletoe from different the host trees has been an object of scientific interest. Qualitative studies of phenolic acids were carried out by Krzaczek (1977) [26] and quantitative studies were conducted by Łuczkiewicz et al. (2001) [28], Vicas et al. (2011) [33], and Stefanucci et al. (2020) [34]. Flavonoid aglycones were studied by Haas et al. (2003) [35], Vicas et al. (2011) [33], and Stefanucci et al. (2020) [34]. The chemical composition of mistletoe was the subject of some publications, but these studies in most cases were focused on *Viscum* species without specifying the host trees from which the plant material was collected. A comparison of the present findings and the literature results of studies of the same mistletoe host tree is shown in Table 8.

The differences in the occurrence of phenolic acids and flavonoid aglycones in the *V. album* species may depend on many factors that should be examined more closely. One of the most likely causes of the qualitative differences is the method used to determine the content of the tested compounds. Łuczkiewicz et al. (2001) and Vicas et al. (2011) determined the compounds using high-performance liquid chromatography, while HPLC in this study was coupled with mass spectrometry, which significantly increased the accuracy and sensitivity of the determination of phenolic substances. In the LC-ESI-MS/MS method, the tested compounds are identified based on not only the retention time but also fragmentation ions. In our research, many other unidentified compounds were found during the analysis, with similar or very similar retention times and even fragmentation ion masses, which indicates a huge diversity in the chemical composition of mistletoe. The influence of other factors should also be investigated, e.g., the time and place of harvesting the plant material, the method of preparation of samples for analysis, the conditions of analysis, and the detectability and content of individual phenolic compounds.

## 3. Materials and Methods

### 3.1. Plant Metarial

To determine the impact of the harvest time on the content and chemical composition of plant material, mistletoe herb (leaves and stems) was collected every four weeks from ten host trees of *Populus nigra* L. located close to Olszowiec, Poland (51o21″ N; 22o13″ E) from February 2016 to April 2017.

To determine the effect of the harvesting place on the composition and antioxidant activity of the plant material, mistletoe herb was harvested from ten apple trees (*Malus domestica* Borkh.) in one orchard from five different places located close to the Lublin region (Poland) in November 2017. Details are shown in Supplementary Table S1.

For analysis of the impact of the host tree species on the chemical composition and antioxidant activity of the plant material, mistletoe leaves and stems were harvested from eleven different host trees: *V. album* subsp. *album* from *Populus nigra* L., *Populus nigra* L. ‘Italica’, *Fraxinus pennsylvanica* Marsh., *Fraxinus exscelsior* L., *Acer platanoides* L., *Acer saccharinum* L., *Malus domestica* Borkh*., Sorbus aucuparia* L., *Tilia cordata* Mill.; *V. album* subsp. *austriacum* from *Pinus sylvestris* L., and *V. album* subsp. *abietis* from *Abies alba* Mill. growing close to the Lublin region (Poland) in winter 2017/2018. In all cases, the plant material from the same host trees was averaged, air-dried at room temperature, and powdered.

### 3.2. Extraction Method

The method of mistletoe extraction was optimized and described previously by Pietrzak et al. (2014).

An amount of 5 g of powdered herb mistletoe (leaves and stems) was extracted using accelerated solvent extractions (ASE). Accelerated solvent extractions (3 times for 15 min each) were conducted in the ASE 150 system from Dionex Corporation (Sunnyvale, CA, USA) at 40 °C with 80% methanol concentrations.

In all cases, the extracts obtained were filtered, evaporated to dryness under vacuum, and lyophilized in the Free Zone 1 apparatus (Labconco, Kansas City, KS, USA). The residue was weighed and redissolved in the same solvent as that used for extraction to obtain stock solutions with a suitable concentration.

All samples were prepared in triplicate.

### 3.3. Total Phenolic (TPC) and Flavonoid Content (TFC)

The total phenolic content (TPC) and total flavonoid content (TFC) were determined using 96-well transparent microplates (Nunclon. Nunc. Roskilde, Denmark) and an Infinite Pro 200F microplate reader (Tecan Group Ltd., Männedorf, Switzerland).

The analysis of total phenolic content was carried out using the modified Folin–Ciocalteu method described earlier by Olech et al. (2012) [36]. Absorbance was read at 680 nm after 20-min incubation. TPC was determined using a standard curve prepared for gallic acid. The results were expressed as mg of gallic acid per 1 g of dry weight of plant material (GAE—gallic acid equivalent).

The total flavonoid content (TFC) was determined according to the modified Lamaison and Carret (1990) method [37,38]. The absorbance was measured at 430 nm after 30-min incubation against a blank methanol-containing solution instead of the test sample. The results were expressed as mg quercetin (Q) per 1 g of dry extract.

### 3.4. Antioxidant Activity Analysis

The antioxidant assay was determined with the DPPH^•^ (2,2-diphenyl−1-picrylhydrazyl) method with some modifications [37,39]. The absorbance was measured at 517 nm after a 30-min incubation. The inhibition curves were prepared and EC_50_ values, defined as the amount of the antioxidant necessary to decrease the initial DPPH^•^ concentration by 50%, were determined.

The results were expressed as mg of dry extract per 1 mg of DPPH^•^.

The antiradical activity was assayed using an improved ABTS^+•^ decolorization assay with modifications [40,41]. The absorbance was measured at 734 nm after a 6-min incubation. The ability of the extract to quench the ABTS^+•^ free radical was determined using the following Equation (1):

Scavenging % = [(AC − AA)/AC] · 100(1)

where AC is the absorbance of the control and AA is the absorbance of the sample.

The results were expressed as Trolox equivalent antioxidant capacity (TEAC) (mM of Trolox per g of dry extract) based on their EC_50_ values.

The Oxygen Radical Absorbance Capacity (ORAC) assay was performed according to the modified method developed by Dienaite et al. (2020) [42,43].

The sample activity was expressed as mM of Trolox per g of dry extract.

All determinations were carried out using the Infinite Pro 200F microplate reader in triplicate.

### 3.5. LC-ESI-MS/MS Analysis

#### 3.5.1. Chromatographic Conditions and Apparatus

Phenolic acids and flavonoid aglycones were determined with reversed-phase high-performance liquid chromatography and electrospray ionization mass spectrometry (LC-ESI-MS/MS).

The Agilent 1200 Series HPLC system (Agilent Technologies, Santa Clara, CA, USA) equipped with a binary gradient solvent pump, a degasser, an autosampler, and a column oven connected to a 3200 QTRAP Mass spectrometer ((Sciex, Redwood City, CA, USA) was used.

The contents of phenolic acids and free flavonoid aglycones were determined with a simple and rapid method using liquid chromatography–electrospray ionization–tandem mass spectrometry. The compounds were separated at 25 °C on a Zorbax SB-C18 column (2.1 × 50 mm, 1.8-μm particle size; Agilent Technologies, Santa Clara, CA, USA).

The contents of phenolic acids were determined with the LC-ESI-MS/MS method described previously by Nowacka et al. (2014) [44] with some modifications.

The determination of flavonoid aglycones was carried out using the LC-ESI-MS/MS method described by Pietrzak et al. (2017) [45] with some modifications.

#### 3.5.2. Optimization of Parameters for the Quantitative Analysis

The 3200 QTRAP MS/MS system and an electrospray ion source in the negative mode were used. The optimal mass spectrometer parameters were determined experimentally and were as follows for the analysis of flavonoid aglycones: curtain gas 20 psi, capillary temperature 500 °C, nebulizer gas 30 psi, and negative ionization mode source voltage −4500 V. The following parameters were used for the analysis of phenolic acids: curtain gas 25 psi, capillary temperature 500 °C, nebulizer gas 60 psi, and negative ionization mode source voltage −4500 V.

Nitrogen was used as the nebulizer and collision gas. The quantitative analysis of the compounds was performed with multiple reaction monitoring (MRM), and Analyst 1.5 software (AB Sciex, Foster City, CA, USA) was used for data acquisition and analysis.

Multiple reaction monitoring (MRM) was used for the quantitative analysis of the compounds. The identified phenolic acids and flavonoid aglycones were quantified based on their peak areas and comparison with the calibration curve for the corresponding standards. Linearity ranges for the calibration curves were specified.

The limits of detection (LOD) and quantification (LOQ) for phenolic acids and flavonoid aglycones were determined at a signal-to-noise ratio of 3:1 and 10:1, respectively, by injecting a series of diluted solutions of known concentrations.

A summary of the optimized parameters for the quantitative analysis of phenolic acids and flavonoid aglycones determined by the LC-ESI-MS/MS method is presented in Supplementary Tables S2 and S3.

### 3.6. Statistical Analysis

All results were expressed as mean ± standard deviation (SD) from three replications. Calculations were performed in STATISTICA 10.0 (StatSoft Poland, Cracow, Poland). The one-way ANOVA test followed by Tukey’s post hoc test was used for statistical analysis of the differences among the obtained data. Significance was accepted at *p* < 0.05.

The results of the spectrophotometric and LC-MS determinations were tested by chemometric analysis. Two methods of cluster analysis were applied: joining (tree clustering) and k-means clustering.

Calculations were performed in STATISTICA 10.0 (StatSoft).

## 4. Conclusions

In conclusion, the period, place of mistletoe harvest, and host tree species significantly affect the content of bioactive components and antiradical properties of extracts obtained from this plant material.

The study has shown that the chemical profile and biological activity of the plant material are closely related to the climatic conditions. The best time to harvest mistletoe during the year is the autumn–winter period. Higher contents of flavonoids and polyphenols and high antioxidant activity were found for extracts obtained from plant material collected in cold weather, in the presence of snow, and in less sunshine.

The qualitative and quantitative composition and biological activity of mistletoe extracts, and hence their practical use, largely depend on the origin of the plant, i.e., the host tree from which the plant was harvested. The chemometric analysis showed that mistletoe collected from conifers (*Viscum album* subsp. *abietis* and *Viscum album* subsp. *austriacum*) is the most advantageous in terms of chemical and antioxidant activity.

The content of secondary plant metabolites is not stable. Factors such as the region/location effect and seasonal variations have an impact on their chemical composition, especially in parasitic plants. It should be emphasized that the present study is the first of such a comprehensive analysis of the influence of climatic factors, place of harvest, and host tree species on the quality of harvested *Viscum* herb. However, there is a need to continue long-term studies in this field.

## Figures and Tables

**Table 1:** Meteorological conditions during harvest time in Olszowiec region based on climate monitoring bulletin Polish.

Sample Name	The Average Air Temperature (°)	Max Snow Depth (cm)	Max Running Time with Snow Cover (days)	The Number of Days with Snow (days)	Monthly Total Precipitation (mm*)*	Sunshine Duration (h)
February’16	−6.1	17	24	27	20	70–80
March’16	5.9	-	-	-	30–40	150–160
April’16	9.3	2	-	1	30–40	180–190
May’16	15.1	-	-	-	30–40	280–300
June’16	16.8	-	-	-	80–90	210–220
July’16	20.3	-	-	-	50–60	280–300
August’16	18.7	-	-	-	50–60	220–240
September’16	14.5	-	-	-	30–40	170–180
September/October’16	11.4	-	-	-	60–70	150–160
October’16	8.2	-	-	-	80–90	120–130
November’16	5.6	-	-	-	20–30	30–40
December’16	−2.5	14	29	30	20–30	20–30
January’17	−3.1	29	22	23	50–60	20–30
February’17	−0.5	25	28	20	40–50	0–20
March’17	−1.8	20	24	26	50–60	110–120
April’17	8.2	27	11	11	40–50	150–160
Winter 2016	−1.3	17	42	53	100–125	150–160
Winter 2017	−2	29	50	81	100–125	70–80

**Table 2:** Spearman correlation coefficient values (X, Y) for the tested parameters.

Test Parameter	R _Spearman_ (X, Y)
TPC and the average air temperature	−0.8355
TPC and maximum snow depth	0.8341
TPC and maximum running time with snow cover	0.8297
TPC and the number of days with snow	0.7698
TFC and the average air temperature	−0.1631
TFC and maximum snow depth	0.3208
TFC and maximum running time with snow cover	0.2231
TFC and the number of days with snow	0.1251
EC_50_ and the average air temperature	0.7466
EC_50_ and maximum snow depth	−0.7768
EC_50_ and maximum running time with snow cover	−0.7444
EC_50_ and the number of days with snow	−0.7178

**Table 3:** Phenolic acid contents (µg per g of dry extract) in extracts from mistletoe collected from *Malus domestica* Borkh. (±SD, *n* = 9).

Compound	*Viscum album* subsp*. album* (from *Malus domestica Borkh.*)				
	T1	T2	T3	T4	T5
Gallic acid	-^b^	-^b^	-^b^	-^b^	-^b^
Protocatechuic acid	70.71 ± 0.57	114.10 ± 2.62	64.60 ± 0.039	107.31 ± 5.37	233.44 ± 10.27
Gentisic acid	-^b^	-^b^	-^b^	-^b^	-^b^
4-hydroxybenzoic acid	trace ^a^	trace ^a^	trace ^a^	27.39 ± 1.10	8.85 ± 0.10
Vanilic acid	trace ^a^	trace ^a^	trace ^a^	trace ^a^	trace ^a^
Caffeic acid	30.10 ± 0.39	21.4 ± 0.13	48.32 ± 0.19	57.91 ± 1.33	50.65 ± 0.25
Syringic acid	46.06 ± 2.07	56.23 ± 0.56	52.87 ± 0.85	72.95 ± 1.31	51.55 ± 0.10
*p*-Coumaric acid	15.05 ± 0.33	11.41 ± 0.40	16.68 ± 0.30	16.60 ± 0.65	15.05 ± 0.38
Ferulic acid	5.59 ± 0.28	7.71 ± 0.11	10.88 ± 0.30	13.49 ± 0.26	10.09 ± 0.24
Salicylic acid	trace ^a^	trace ^a^	0.72 ± 0.01	2.79 ± 0.01	0.81 ± 0.04
Veratric acid	-^b^	-^b^	-^b^	-^b^	-^b^
Synapic acid	28.14 ± 0.06	30.24 ± 0.42	24.40 ± 1.12	68.87 ± 1.38	15.05 ± 0.20
*m*-Coumaric acid	-^b^	-^b^	-^b^	-^b^	-^b^
Rosmarinic acid	trace ^a^	trace ^a^	trace ^a^	trace ^a^	trace ^a^
TOTAL	195.65	241.09	218.47	367.31	385.49

^a^ trace—trace amounts; ^b^—not detected.

**Table 4:** Total phenolic content (TPC; mg GA per g of dry extract), flavonoid content (TFC; mg Q per g of dry extract) and antioxidant activity (EC50; mg dry extract per mg of DPPH^•^) of extracts from mistletoe collected from *Malus domestica* Borkh.

	*Viscum album* subsp*. album* (from *Malus domestica Borkh.*)				
	T1	T2	T3	T4	T5
TPC	34.800 ± 0.728	48.628 ± 1.316	36.655 ± 1.666	37.216 ± 0.190	33.789 ± 1.606
TFC	13.786 ± 0.257	12.662 ± 0.092	11.722 ± 0.235	13.571 ± 0.155	12.977 ± 0.026
EC_50_	4.608 ± 0.190	3.824 ± 0.073	3.753 ± 0.094	3.92 ± 0.086	4.136 ± 0.095

**Table 5:** Total phenolic content (TPC), flavonoid content (TFC), antioxidant activity (DPPH^•^ scavenging assay, antiradical capacity determination with ABTS^+•^, Oxygen Radical Absorbance Capacity (ORAC) assay) and efficiency of extraction (EE) (g of dry extract obtained from 1 g raw material) in different mistletoe extracts. Values are presented in mean ± standard deviation (*n* = 9) and evaluated by one-way ANOVA test (post test: Duncan test). Different superscript letters (^a–g^) in the same rows denotes significant differences at *p* < 0.05.

Samples	EE (%)	TPC (mg GA/g Dry Extract)	TFC (mg Q/g Dry Extract)	Antioxidant Acticity		
				EC_50_ (mg Dry Extract/mg DPPH^•^)	TEAC (mM Trolox/g Dry Extract)	ORAC (mM Trolox/g Dry Extract)
*Viscum album* subsp. *album*						
*Malus domestica* Borkh	0.172	38.22 ^a^ ± 0.73	12.94 ^adef^ ± 0.26	4.05 ^abc^ ± 0.19	0.09 ^a^ ± 0.02	1.54 ^a^ ± 0.05
*Populus nigra* L.	0.181	38.53 ^a^ ± 0.23	16.36 ^bc^ ± 0.41	2.37 ^ab^ ± 0.09	0.18 ^b^ ± 0.04	1.96 ^b^ ± 0.09
*Populus nigra* L. ‘Italica’	0.169	38.09 ^a^ ± 0.96	16.04 ^bcf^ ± 0.28	2.88 ^ab^ ± 0.08	0.15 ^b^ ± 0.01	1.78 ^a^ ± 0.02
*Crataegus monogyna* Jacq.	0.158	38.05 ^a^ ± 1.07	12.09 ^ad^ ± 0.51	2.92 ^ab^ ± 0.12	0.17 ^b^ ± 0.01	1.88 ^b^ ± 0.05
*Sorbus aucuparia* L.	0.139	38.73 ^a^ ± 0.26	12.86 ^ade^ ± 0.21	4.35 ^a^ ± 0.08	0.07 ^a^ ± 0.03	1.41 ^a^ ± 0.09
*Fraxinus pensylvanica* Marsh.	0.170	31.49 ^b^ ± 0.86	14.19 ^aef^ ± 0.09	2.99 ^ab^ ± 0.07	0.15 ^b^ ± 0.02	1.91 ^b^ ± 0.03
*Fraxinus exscelsior* L.	0.168	30.56 ^bc^ ± 1.76	14.56 ^acef^ ± 0.35	4.21^ab^ ± 0.11	0.08 ^a^ ± 0.02	1.25 ^a^ ± 0.06
*Acer platanoides* L.	0.143	33.49 ^d^ ± 0.09	13.14 ^adef^ ± 0.47	2.78 ^ab^ ± 0.15	0.14 ^b^± 0.01	1.92 ^b^ ± 0.07
*Acer saccharinum* L.	0.158	32.33 ^bcd^ ± 0.07	13.21^adef^ ± 0.52	2.14 ^ab^ ± 0.09	0.19 ^b^ ± 0.03	2.02 ^b^ ± 0.08
*Tilia cordata* Mill.	0.166	34.91 ^d^ ± 0.23	11.89 ^ad^ ± 0.24	2.79 ^ab^ ± 0.12	0.12 ^b^ ± 0.02	1.84 ^b^ ± 0.09
*V. album* subsp. *abietis*						
*Abies alba* Mill.	0.164	56.75 ^e^ ± 1.31	10.11 ^g^ ± 0.42	2.57 ^ab^ ± 0.10	0.21 ^b^ ± 0.08	1.95 ^b^ ± 0.10
*V. album* subsp. *austriacum*						
*Pinus sylvestris* L.	0.190	39.11 ^a^ ± 1.21	16.90 ^bc^ ± 0.32	2.56 ^ab^ ± 0.11	0.19 ± 0.07	2.01 ± 0.10

**Table 6:** Phenolic acids and flavonoid aglycones contents (µg/g dry extract) in extracts from *Viscum album* subsp. *album* collected from different host trees (average, *n* = 9).

Compound	*Viscum album* subsp. *album*									
	*Malus domestica Borkh*	*Populus nigra* L.	*Populus nigra* L. ‘Italica’	*Crataegus monogyna* Jacq.	*Sorbus aucuparia* L.	*Fraxinus pensylva-nica* Marsh.	*Fraxinus exscelsior L.*	*Acer platanoides L.*	*Acer saccharinum L.*	*Tilia cordata* Mill.
Phenolic acids										
Gallic acid	-^b^	-^b^	-^b^	-^b^	trace ^a^	trace ^a^	46.62 ± 0.15	-^b^	-^b^	33.71 ± 0.15
Protocatechuic acid	118.03 ± 0.86	31.30 ± 0.05	6.77 ± 0.08	72.97 ± 0.78	25.80 ± 0.20	133.27 ± 1.20	203.48 ± 0.15	105.08 ± 1.35	trace ^a^	138.17 ± 1.17
Gentisic acid	-^b^	trace ^a^	trace ^a^	-^b^	-^b^	-^b^	trace ^a^	-^b^	-^b^	trace ^a^
4-hydroxybenzoic acid	18.12 ± 0.24	37.48 ± 0.25	31.70 ± 0.17	trace ^a^	56.35 ± 0.35	trace ^a^	1.28 ± 0.01	11.73 ± 0.11	21.45± 0.01	18.65 ± 0.25
Vanilic acid	trace ^a^	72.90 ± 0.15	56.69 ± 0.15	trace ^a^	46.95 ± 0.15	trace ^a^	trace ^a^	trace ^a^	36.59± 0.15	34.71 ± 0.15
Caffeic acid	41.67 ± 0.74	43.64 ± 0.35	33.98 ± 0.24	19.86 ± 0.13	36.27 ± 0.24	17.26 ± 0.15	58.61 ± 0.89	33.44 ± 0.25	4.57 ± 0.05	45.03 ± 0.45
Syringic acid	55.93 ± 0.55	129.49 ± 1.23	234.60 ± 4.54	49.87 ± 0.41	154.20 ± 1.35	63.71 ± 0.55	41.84 ± 0.36	86.80 ± 0.08	79.50± 0.27	64.55 ± 0.54
*p*-Coumaric acid	14.96 ± 0.15	16.62 ± 0.17	28.80 ± 0.15	17.70 ± 0.28	18.47 ± 0.27	trace ^a^	25.71 ± 0.08	17.70 ± 0.09	25.30± 0.30	23.92 ± 0.27
Ferulic acid	9.55 ± 0.07	15.48± 0.05	12.21 ± 0.02	5.99 ± 0.06	8.43 ± 0.04	7.45 ± 0.01	6.78 ± 0.10	7.99± 0.10	5.29 ± 0.04	7.75 ± 0.07
Salicylic acid	1.44 ± 0.01	8.48 ± 0.06	9.62 ± 0.06	0.32 ± 0.02	3.23 ± 0.02	0.28 ± 0.02	1.45 ± 0.01	0.70± 0.01	2.67 ± 0.01	1.56± 0.01
Veratric acid	-^b^	-^b^	-^b^	-^b^	-^b^	-^b^	-^b^	-^b^	-^b^	-^b^
Synapic acid	33.34 ± 0.40	52.08 ± 0.45	84.94± 0.34	26.40± 0.29	50.71 ± 0.51	38.2 ± 0.35	101.60± 1.20	88.83 ± 1.15	1.94 ± 0.01	59.65 ± 0.50
*m*-Coumaric acid	-^b^	-^b^	-^b^	-^b^	-^b^	-^b^	-^b^	-^b^	-^b^	-^b^
Rosmarinic acid	trace ^a^	trace ^a^	trace ^a^	trace ^a^	trace ^a^	trace ^a^	trace ^a^	trace ^a^	trace ^a^	trace ^a^
TOTAL of phenolic acids	281.60	419.32	495.23	193.11	376.91	260.17	480.59	344.27	172.02	327.28
Flavonoid aglycones										
Taxifolin	-^b^	-^b^	-^b^	-^b^	-^b^	-^b^	-^b^	-^b^	-^b^	-^b^
Myricetin	1.91 ± 0.15	-^b^	trace ^a^	trace ^a^	0.10 ± 0.01	trace ^a^	trace ^a^	trace ^a^	0.5 ± 0.01	trace ^a^
Morin	-^b^	-^b^	-^b^	-^b^	-^b^	-^b^	-^b^	-^b^	-^b^	-^b^
Eriodictyol	1.73 ± 0.01	-^b^	3.20 ± 0.02	1.16 ± 0.00	trace ^a^	5.06 ± 0.01	5.90 ± 0.01	-^b^	-^b^	6.55 ± 0.01
Luteolin	-^b^	-^b^	trace ^a^	-^b^	trace ^a^	trace ^a^	-^b^	trace ^a^	-^b^	1.67 ± 0.15
Quercetin	1.88 ± 0.01	-^b^	-^b^	-^b^	-^b^	-^b^	-^b^	-^b^	-^b^	-^b^
3-O-Methylquercetin	2.64 ± 0.05	2.42 ± 0.02	10.39± 0.01	12.43± 0.17	1.16 ± 0.01	5.04 ± 0.00	5.49 ± 0.00	1.50± 0.00	1.28± 0.05	8.97 ± 0.05
Apigenin	trace ^a^	30.86± 0.15	trace ^a^	trace ^a^	trace ^a^	trace ^a^	-^b^	57.32 ± 0.15	trace ^a^	trace ^a^
Naringenin	0.43 ± 0.15	trace ^a^	1.99 ± 0.15	trace ^a^	trace ^a^	1.16 ± 0.15	trace ^a^	3.95 ± 0.15	trace ^a^	1.15 ± 0.15
Kaempferol	-^b^	-^b^	-^b^	-^b^	-^b^	-^b^	-^b^	trace ^a^	-^b^	-^b^
Isorhamnetin	1.83 ± 0.15	-^b^	-^b^	-^b^	-^b^	-^b^	-^b^	-^b^	trace ^a^	trace ^a^
Rhamnetin	12.5 ± 0.01	-^b^	trace ^a^	-^b^	trace ^a^	trace ^a^	-^b^	-^b^	trace ^a^	trace ^a^
Chrysin	-^b^	-^b^	-^b^	-^b^	-^b^	-^b^	-^b^	-^b^	-^b^	-^b^
Sakuranetin	2.22 ± 0.02	-^b^	-^b^	-^b^	trace ^a^	trace ^a^	-^b^	-^b^	trace ^a^	-^b^
Prunetin	-^b^	-^b^	-^b^	-^b^	-^b^	-^b^	-^b^	-^b^	-^b^	-^b^
Rhamnazin	32.08 ± 0.30	trace ^a^	-^b^	trace ^a^	11.56 ± 0.20	trace ^a^	-^b^	trace ^a^	7.86 ± 0.17	3.64 ± 0.15
TOTAL of flavonoid aglycones	57.14	33.28	15.58	13.59	26.41	11.26	11.39	62.77	9.64	21.98

^a^ trace—trace amounts; ^b^—not detected.

**Table 7:** Phenolic acids and flavonoid aglycones contents (µg/g dry extract) in extracts from *Viscum album* subsp. *abietis* and *austriacum* (average, *n* = 9).

Compound	*V. album* subsp. *abietis*	*V. album* subsp. *austriacum*
	*Abies alba* Mill.	*Pinus sylvestris* L.
Phenolic acids		
Gallic acid	-^b^	-^b^
Protocatechuic acid	114.29 ± 1.05	17.90 ± 0.20
Gentisic acid	trace ^a^	trace ^a^
4-hydroxybenzoic acid	164.91 ± 4.95	23.14 ± 0.25
Vanilic acid	95.04 ± 0.15	81.24 ± 0.15
Caffeic acid	48.82 ± 0.47	53.71 ± 0.56
Syringic acid	163.91 ± 1.47	167.62 ± 1.62
*p*-Coumaric acid	44.11 ± 0.17	42.76 ± 0.41
Ferulic acid	5.31 ± 0.04	9.81 ± 0.08
Salicylic acid	1.88 ± 0.02	5.24 ± 0.04
Veratric acid	-^b^	-^b^
Synapic acid	17.31 ± 0.07	11.68 ± 0.11
*m*-Coumaric acid	-^b^	-^b^
Rosmarinic acid	trace ^a^	trace ^a^
TOTAL of phenolic acids	601.45	403.29
Flavonoid aglycones		
Taxifolin	-^b^	-^b^
Myricetin	trace ^a^	0.12 ± 0.00
Morin	-^b^	-^b^
Eriodictyol	-^b^	0.15 ± 0.00
Luteolin	-^b^	-^b^
Quercetin	-^b^	0.21 ± 0.02
3-*O*-Methylquercetin	0.11 ± 0.02	1.41 ± 0.01
Apigenin	0.43 ± 0.02	trace ^a^
Naringenin	1.75 ± 0.01	1.41 ± 0.00
Kaempferol	-^b^	1.07 ± 0.01
Isorhamnetin	-^b^	2.44 ± 0.01
Rhamnetin	-^b^	3.71 ± 0.02
Chrysin	-^b^	-^b^
Sakuranetin	-^b^	14.44 ± 0.17
Prunetin	-^b^	-^b^
Rhamnazin	-^b^	31.12 ± 0.10
TOTAL of flavonoid aglycones	2.29	56.08

^a^ trace—trace amounts; ^b^—not detected.

**Table 8:** Summary and comparison of the qualitative results of phenolic acids and flavonoid aglycones of mistletoe leaf extracts obtained in this study and included in the literature. Marking: “+”—compound present in the tested sample; “-” no presence in the tested sample; “NT”—not tested.

*Viscum album* L./host tree		Own research	Łuczkiewicz et al. 2001 [28]	Haas et al. 2003 [35]	Vicas et al. 2011 [33]	Krzaczek 1977 [26]	Stefanucci et al. 2020 [34]
Gallic acid							
*Viscum album* subsp*. album*	*Acer platanoides* L.	-	+	NT	NT	NT	NT
	*Fraxinus excelsior* L.	+	NT	NT	-	NT	NT
	*Populus nigra* L.	-	+	NT	-	NT	NT
	*Malus domestica* Borkh.	-	+	NT	-	NT	NT
	*Sorbus aucuparia* L.	trace	+	NT	NT	NT	NT
Gentisic acid							
*Viscum album* subsp*. album*	*Acer platanoides* L.	-	+	NT	NT	NT	NT
	*Populus nigra* L.	trace	+	NT	-	NT	NT
	*Malus domestica* Borkh.	-	+	NT	-	NT	NT
	*Sorbus aucuparia* L.	-	+	NT	NT	NT	NT
Syringic acid							
*Viscum album* subsp*. album*	*Populus nigra* L.	+	trace	NT	-	+	NT
Vanilic acid							
*Viscum album* subsp*. album*	*Acer platanoides* L.	trace	-	NT	NT	NT	NT
	*Populus nigra* L.	+	-	NT	NT	+	NT
	*Malus domestica* Borkh.	trace	+	NT	NT	+	NT
	*Sorbus aucuparia* L.	trace	-	NT	NT	NT	NT
Salicylic acid							
*Viscum album* subsp*. album*	*Acer platanoides* L.	+	-	NT	NT	NT	NT
*p*-Coumaric acid							
*Viscum album* subsp*. album*	*Populus nigra* L.	+	+	NT	-	+	NT
	*Malus domestica* Borkh.	+	+	NT	-	+	NT
Ferulic acid							
*Viscum album* subsp*. album*	*Malus domestica* Borkh.	+	-	NB	+	+	NT
*Viscum album* subsp. *abietis*		+	NT	NT	NT	Free acid - No-free acid +	NT
*Viscum album* subsp*. austriacum*		+	NT	NT	NT	Free acid - No-free acid +	NT
Synapic acid							
*Viscum album* subsp*. album*	*Acer platanoides* L.	+	-	NT	NT	NT	NT
	*Populus nigra* L.	+	-	NT	+	Free acid - No-free acid +	NT
	*Malus domestica* Borkh.	+	trace	NB	+	Free acid - No-free acid +	NT
	*Sorbus aucuparia* L.	+	-	NT	NT	NB	NT
*Viscum album* subsp. *abietis*		+	NT	NT	NT	Free acid - No-free acid +	NT
*Viscum album* subsp*. austriacum*		+	NT	NT	NT	Free acid - No-free acid +	+
Veratric acid							
*Viscum album* subsp*. album*	*Malus domestica* Borkh.	-	+	NT	NT	NT	NT
	*Sorbus aucuparia* L.	-	+	NT	NT	NT	NT
Rosmarinic acid							
*Viscum album* subsp*. album*	*Acer platanoides* L.	trace	+	NT	NT	NT	NT
	*Populus nigra* L.	trace	-	NT	+	NT	NT
	*Sorbus aucuparia* L.	trace	-	NT	NT	NT	NT
Quercetin							
*Viscum album* subsp*. album*	*Populus nigra* L.	-	NT	NT	+	NT	NT
	*Fraxinus excelsior* L.	-	NT	NT	+	NT	NT
Kaempferol							
*Viscum album* subsp*. album*	*Fraxinus excelsior* L.	-	NT	NT	+	NT	NT
Naringenin							
*Viscum album* subsp. *abietis*		+	NT	-	NT	NT	NT
*Viscum album* subsp*. austriacum*		+	NT	NT	NT	NT	+
Rhamnetin							
*Viscum album* subsp. *abietis*		-	NT	+	NT	NT	NT
*Viscum album* subsp*. austriacum*		+	NT	-	NT	NT	NT
Isorhamnetin							
*Viscum album* subsp*. austriacum*		+	NT	-	NT	NT	+
Rhamnazin							
*Viscum album* subsp. *abietis*		-	NT	+	NT	NT	NT
*Viscum album* subsp*. austriacum*		+	NT	-	NT	NT	NT

## Data Availability

Data sharing not applicable.

## Supplementary Materials

The following are available online, Figure S1: graphic division of mistletoe herb extracts from *Malus domestica* Borkh. for two clusters using the k-medium method (in terms of phenolic acids content and spectrophotometric analysis of TPC, TFC and antioxidant activities), Figure S2: the chromatogram in MRM mode of flavonoid aglycones in *Viscum austriacum* herb extract: 1- eriodictyol; 2- 3-*O*-methylquercetin; 3- quercetin; 4- naringenin; 5- kaempferol; 6-isorhamnetin; 7- rhamnetin; 8- sakuranetin, Figure S3: the chromatogram in MRM mode of phenolic acids in *Viscum abietis* herb extract: 1- protocatechuic acid; 2- 4-hydroxybenzoic acid; 3- salicylic acid; 4- caffeic acid; 5- syringic acid; 6- *p*-coumaric acid; 7- ferulic acid. Table S1: the harvesting place of mistletoe herb from apple trees (*Malus domestica* Borkh.), Table S2: summary of optimized parameters for the quantitative analysis of phenolic acids and flavonoid aglycones, Table S3: analytical parameters of LC-MS/MS quantitative method for determination of phenolic acids and flavonoid aglycones.

## Sample Availability

Samples of the compounds are available from the authors.
