# Supplementary material for: Impact of Harvest Conditions and Host Tree Species on Chemical Composition and Antioxidant Activity of Extracts from Viscum album L
Source: Molecules. 2021 Jun 19;26(12):3741. doi: 10.3390/molecules26123741 (PMC8233733; doi:10.3390/molecules26123741)
Supplement: Supplementary file 1 [file molecules-26-03741-s001.zip › molecules-1227922-supplementary.pdf]

Supplementary

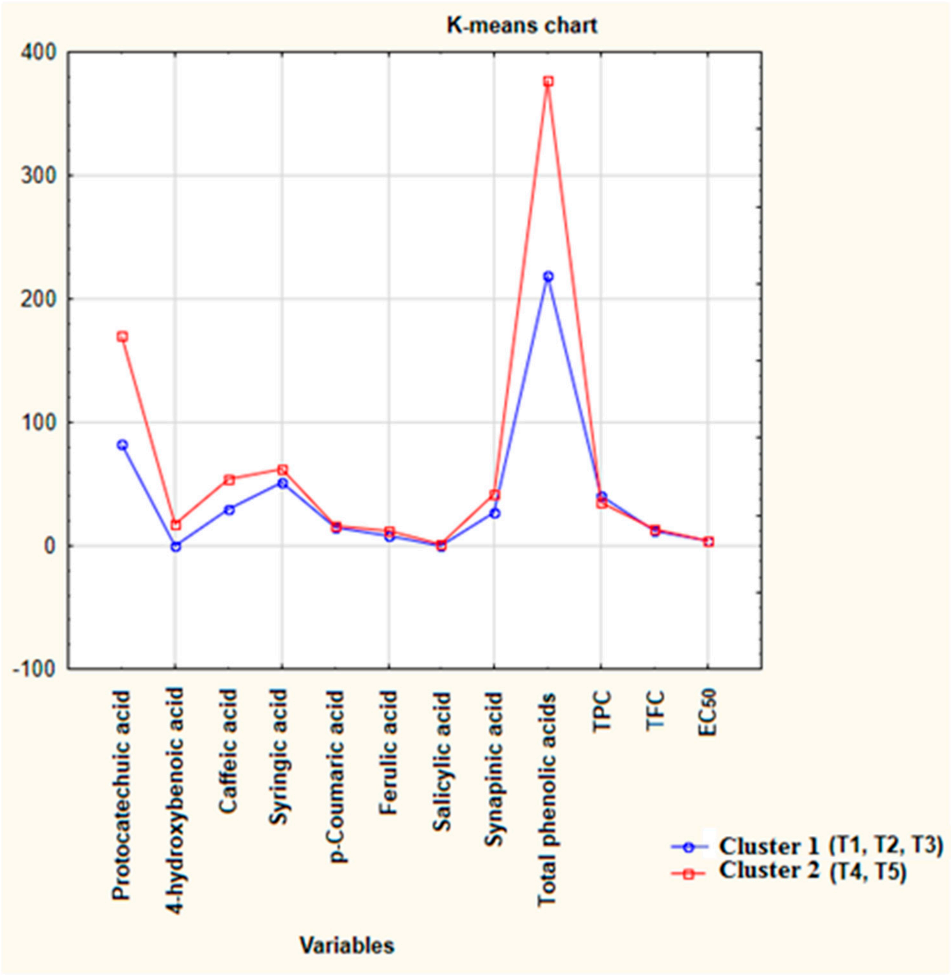

Figure S1. Graphic division of mistletoe herb extracts from *Malus domestica* Borkh. for two clusters using the k-medium method (in terms of phenolic acids content and spectrophotometric analysis of TPC, TFC and antioxidant activities).

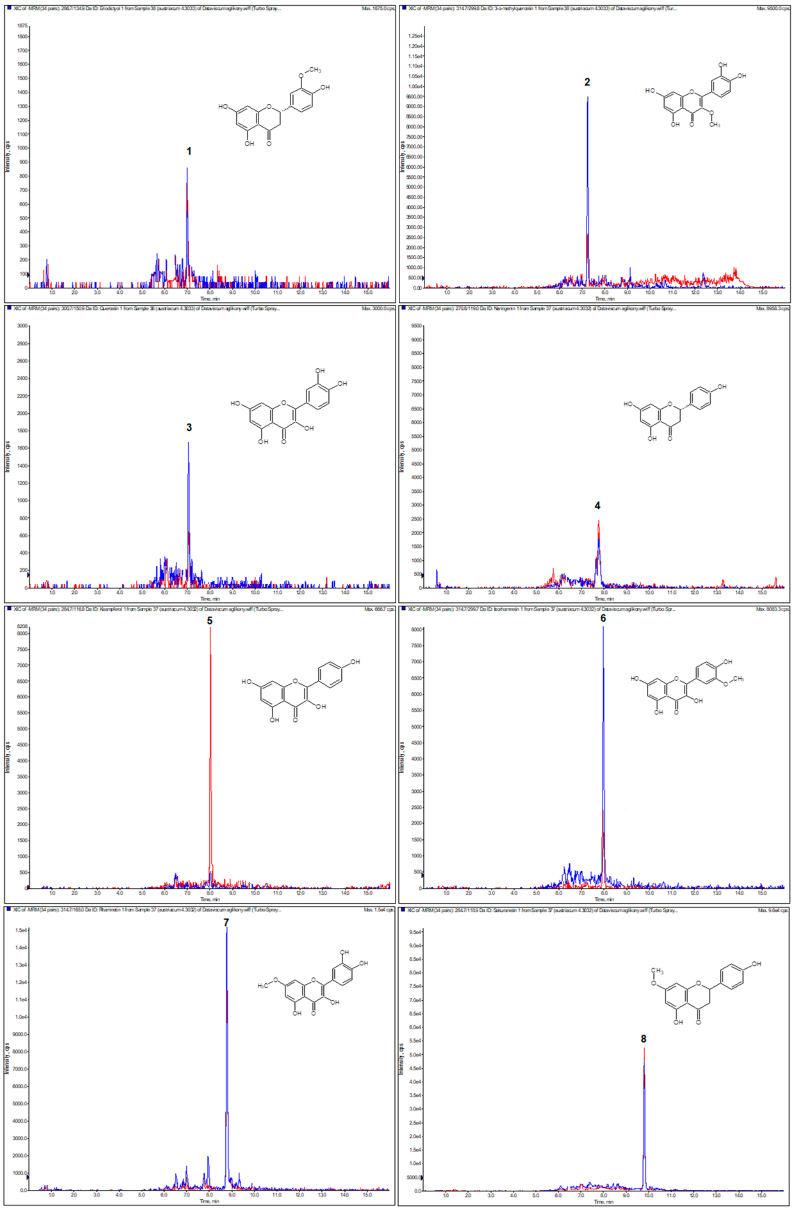

Figure S2. The chromatogram in MRM mode of flavonoid aglycones in *Viscum austriacum* herb extract: 1- eriodictyol; 2- 3-*O*-methylquercetin; 3- quercetin; 4- naringenin; 5- kaempferol; 6-isorhamnetin; 7- rhamnetin; 8- sakuranetin.

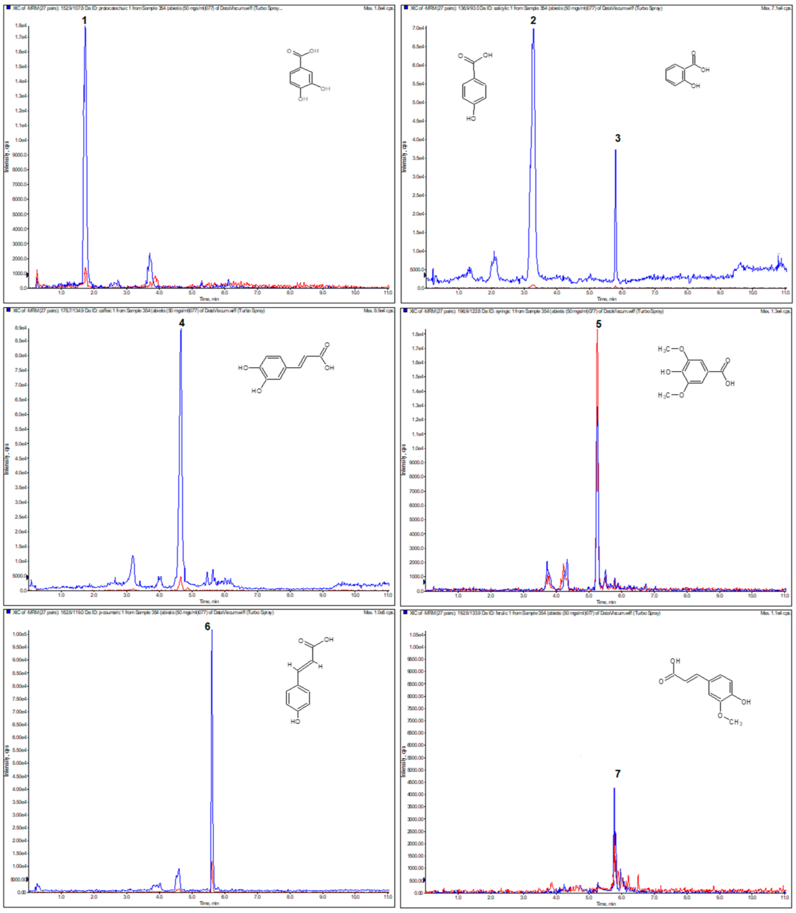

Figure S3. The chromatogram in MRM mode of phenolic acids in *Viscum abietis* herb extract: 1- protocatechuic acid; 2- 4-hydroxybenzoic acid; 3- salicylic acid; 4- caffeic acid; 5- syringic acid; 6- *p*-coumaric acid; 7- ferulic acid.

Table S1. The harvesting place of mistletoe herb from apple trees (*Malus domestica* Borkh.)

Plant material	Host tree	Harvesting location (GPS data)	Time of harvesting	Symbol (number of tested samples)
Viscum album L. subsp. *album*	*Malus domestica* Borkh.	51°18′13′′N 22°23′44′′E	November 2017	T1 (10 samples)
		51°19′57′′N 22°21′35′′E		T2 (10 samples)
		51°17′07′′N 22°18′19′′E		T3 (10 samples)
		51°01′55,91′′N 22°40′36,08′′E		T4 (10 samples)
		51°01′55,91′′N 22°40′36,08′′E		T5 (10 samples)

Table S2. Summary of optimized parameters for the quantitative analysis of phenolic acids and flavonoid aglycones.

**Compound**	**Retention time [min]**	**Q1/Q3 [m/z]**	**DP [V]**	**EP [V]**	**CEP [V]**	**CE [eV]**	**CXP [V]**

**Phenolic acids**							
Gallic acid	0.74	168.7/78.9	-35	-3	-12	-36	0
		168.7/124.9	-35	-3	-12	-14	0
Protocatechuic acid	1.70	152.9/80.9	-55	-1	-10	-26	0
		152.9/107.8	-55	-1	-10	-38	0
Gentisic acid	2.70	352.9/80	-70	-4	-16	-110	0
		352.9/96.9	-70	-4	-16	-52	0
4-Hydroxybenzoic acid	3.26	136.8/92.9	-30	-7	-10	-18	0
Vanilic acid	4.49	166.8//107.9	-35	-4	-12	-18	0
		166.8/123	-35	-4	-12	-12	0
Caffeic acid	4.65	178.7/88.9	-30	-6.5	-12	-46	0
		178.7/134.9	-30	-6.5	-12	-16	0
Syringic acid	5.26	196.9/122.8	-30	-9	-12	-24	0
		196.9/181.9	-30	-9	-12	-12	-2
4-Hydroxycinnamic acid (*p*-coumaric acid)	5.60	162.8/93	-30	-8	-12	-44	0
		162.8/119	-30	-8	-12	-14	0
Ferulic acid	5.77	192.8/133.9	-25	-11.5	-14	-16	0
		192.8/177.9	-25	-11.5	-14	-12	-2
Salicylic acid	5.80	136.9/75	-35	-4	-10	-48	0
		136.9/93	-35	-4	-10	-16	-2
3,4-Dimethoxybenzoic acid (Veratric acid)	5.80	180.7/121.9	-35	-6	-14	-18	0
		180.7/136.9	-35	-6	-14	-12	0
Sinapic acid	5.81	222.8/121	-35	-8.5	-10	-36	0
		222.8/148.9	-35	-8.5	-10	-20	0
3-Hydroxycinnamic acid (*m*-coumaric acid)	5.82	162.8/91	-35	-4.5	-12	-36	0
		162.8/119	-35	-4.5	-12	-14	0
Rosmarinic acid	5.97	358.7/132.6	-50	-5	-26	-44	0
		358.7/160.8	-50	-5	-26	-20	-2
**Flavonoid aglycones**							
Taxifolin	6.17	302.7/124.9	-45	-3.5	-18	-26	0
		302.7/284.8	-45	-3.5	-18	-14	-4
Myricetin	6.47	316.7/136.9	-55	-9	-14	-32	0
		316.7/150.9	-55	-9	-14	-26	0
Morin	6.82	300.7/124.9	-50	-3.5	-20	-24	0
		300.7/106.9	-50	-3.5	-20	-30	0
Eriodictyol	7.07	286.7/134.9	-45	-6	-12	-32	0
		286.7/150.9	-45	-6	-12	-18	-2
Luteolin	7.08	284.7/132.9	-75	-9	-18	-38	0
		284.7/150.9	-75	-9	-18	-26	0
Quercetin	7.13	300.7/150.9	-60	-2.5	-12	-26	0
		300.7/178.8	-60	-2.5	-12	-20	-2
3-*O*-Methylquercetin	7.32	314.7/299.8	-55	-9.5	-22	-18	-4
		314.7/270.8	-55	-9.5	-22	-26	-4
Apigenin	7.77	268.8/117	-70	-9.5	-12	-44	0
		268.8/106.8	-70	-9.5	-12	-34	0
Naringenin	7.81	270.8/119	-50	-11.5	-12	-34	0

		270.8/150.9	-50	-11.5	-12	-22	0
Kaempferol	7.94	284.7/116.8	-70	-5	-12	-46	0
		284.7/93	-70	-5	-12	-52	0
Isorhamnetin	8.09	314.7/299.7	-65	-2.5	-26	-20	-4
		314.7/150.9	-65	-2.5	-26	-30	0
Rhamnetin	8.85	314.7/165	-60	-5.5	-18	-24	0
		314.7/120.9	-60	-5.5	-18	-36	0
Chrysin	9.83	252.8/208.9	-80	-10	-14	-22	-2
		252.8/142.9	-80	-10	-14	-26	0
Sakuranetin	9.89	284.7/118.9	-60	-5.5	-12	-34	0
		284.7/164.8	-60	-5.5	-12	-20	-2
Prunetin	10.18	282.8/267.7	-55	-12	-18	-20	-4
		282.8/238.7	-55	-12	-18	-26	-2
Rhamnazin	10.31	328.7/270.8	-70	-3	-28	-26	-2
		328.7/313.8	-70	-3	-28	-14	-4

Table S3. Analytical parameters of LC-MS/MS quantitative method for determination of phenolic acids and flavonoid aglycones.

Compound	R^2	LOD [ng/mL]	LOQ [ng/ mL]	Linearity range [ng/ mL]
**Phenolic acids**				
Gallic acid	0.9990	50	100	100-10000
Protocatechuic acid	0.9975	10	20	25-25000
Gentisic acid	0.9993	8	15	25-25000
4-Hydroxybenzoic acid	0.9972	50	100	100-5000
Vanilic acid	0.9999	100	200	200-50000
Caffeic acid	0.9975	40	85	100-5000
Syringic acid	0.9997	50	100	100-50000
4-Hydroxycinnamic acid (*p*-coumaric acid)	0.9985	10	25	50-2500
Ferulic acid	0.9995	10	25	25-5000
Salicylic acid	0.9985	10	20	20-700
3,4-Dimethoxybenzoic acid (Veratric acid)	0.9978	400	700	500-25000
Sinapic acid	0.9984	7	25	25-5000
3-Hydroxycinnamic acid (*m*-coumaric acid)	0.9990	20	50	50-2500
Rosmarinic acid	0.9980	5	10	25-25000
**Flavonoid aglycones**				
Taxifolin	0.9952	20	50	50-4 500
Myricetin	0.9958	2	5	10-3 500
Morin	0.9971	2	5	10-4 000
Eriodictyol	0.9979	5	15	15-4 000
Luteolin	0.9967	20	40	40-5 000

Quercetin	0.9980	3	10	30-3 000
3-*O*-Methylquercetin	0.9972	1	2	10-3 000
Apigenin	0.9970	2	5	15-6 500
Naringenin	0.9978	25	30	30-3 100
Kaempferol	0.9974	20	30	30-20 000
Isorhamnetin	0.9976	10	25	40-40 000
Rhamnetin	0.9981	2	5	6-625
Chrysin	0.9964	25	40	40-2 500
Sakuranetin	0.9963	30	40	70-7 000
Prunetin	0.9968	25	50	100-10 000
Rhamnazin	0.9969	25	50	70-7 000
